# Altered Salivary Alpha-Amylase Secretion in Patients with Ulcerative Colitis

**DOI:** 10.1155/2018/4203737

**Published:** 2018-05-24

**Authors:** Zhuoni Xu, Baoping Wei, Yanting Qiu, Tao Zhang

**Affiliations:** ^1^Ruikang Hospital of Guangxi Traditional Chinese Medical University, Nanning, Guangxi, China; ^2^The First Affiliated Hospital of Guangxi Traditional Chinese Medical University, Nanning, Guangxi, China

## Abstract

**Purpose:**

Patients with ulcerative colitis (UC) frequently present with psychological disturbances as well as dysfunctions of autonomic nervous system (ANS). Salivary alpha-amylase (sAA) secretion is predominantly controlled by sympathetic nervous activity, while salivary fluid secretion is by parasympathetic nervous activity. Thus, it is speculated that alterations of salivary secretion may be addressed in UC populations.

**Methods:**

Thirty-five UC patients as well as 32 age- and sex-matched healthy controls were enrolled. Saliva samples before and after citric acid stimulation were collected from each participant, and salivary flow rate (SFR) was calculated accordingly. Western blotting and quantitative PCR were applied to measure the sAA level and sAA gene (*AMY1*) copy number, respectively. The psychological disorders, anxiety and depression, were evaluated by the scoring system of Hospital Anxiety and Depression Scale (HADS) for each participant.

**Results:**

We observed robustly increased prevalence of anxiety (*p* < 0.001) as well as depression (*p* < 0.001) in UC patients relative to controls. Interestingly, we detected elevated basal (*p* = 0.015) and stimulated (*p* = 0.021) sAA levels in the UC populations compared to controls. However, no differences were found for basal (*p* = 0.643) or stimulated (*p* = 0.402) SFR between the two study groups. Besides, *AMY1* gene copy number was comparable between UC patients and controls.

**Conclusions:**

Our results reveal an overactivity of the sympathetic nervous system and a normal activity of the parasympathetic nervous system in the UC population.

## 1. Introduction

Ulcerative colitis (UC) is a major form of inflammatory bowel disease and is characterized by chronic inflammation of the colon and rectum and by alternating periods of flares and remissions. Patients typically present with abdominal pain, diarrhea, and blood in the stools [1]. UC has emerged as a global disease because its incidence and prevalence are increasing with time and in different regions around the world [2]. It is a lifelong illness with significant effects on the quality of life and is also associated with increased risk of colorectal cancer due to its presence of local chronic inflammation [3, 4]. To date, the pathogenesis of UC remains elusive.

UC has been long claimed to be of psychosomatic origin since a half decade ago. It is been widely documented that UC patients apparently have higher prevalence of psychiatric disturbances than normal controls [5–7]. For example, Magni and colleagues detected a prevalence of 62% of psychiatric disorders, mainly anxiety and minor depression, in the UC population, which was much higher than the controls [7]. Some investigators have noted that the psychological disorders might probably be of a consequence of disease activity [8]. Yet others believed that psychological stress was able to influence disease course and exacerbations [9, 10].

The autonomic nervous system (ANS) is an important regulator of intestinal inflammatory activity in an animal model [11]. Specifically, the sympathetic activity is considered to have proinflammatory properties in the acute phase of inflammation [12], while the parasympathetic division has anti-inflammatory properties via the vagus nerve [13]. There is growing body of evidence that the ANS abnormalities may play a role in the UC pathophysiology. For example, alterations of the sympathetic nervous activity were detected to sustain local inflammatory mechanisms in the UC animal model [14], and previous studies had demonstrated altered ANS activities in UC patients [15–17]. More interestingly, an accumulating body of evidence suggests that the psychological disorders are closely associated with the altered ANS activity and that the ANS dysfunction is one of the pathophysiological links between the psychological disorders and a series of somatic diseases [18–23], including UC [24].

Salivary alpha-amylase (sAA) is one of the most abundant proteins in saliva [25]. Genetically, sAA production is mainly determined by the copy number of the sAA gene, *AMY1* [26]. The sAA level positively correlates with *AMY1* copy number in adults [27, 28]. *AMY1* gene shows extensive variations in the copy number, with a range from 2 to 15 diploid copies [29, 30]. sAA secretion is predominantly controlled by the sympathetic nervous system via the release of noradrenaline from sympathetic neurons [31]. Mechanically, after release, noradrenaline binds to the beta-adrenergic receptor on the acinar cells of the salivary glands, which induces an elevation of intracellular cyclic adenosine monophosphate followed by sAA secretion from the acinar cells [32–34]. Recently, sAA has been implicated as a noninvasive biomarker for the activity of the sympathetic nervous system [31]. Unlike sAA secretion, salivary fluid secretion is mainly regulated by the parasympathetic nervous system. Mechanistically, the cholinergic parasympathetic nerves release acetylcholine that binds to the M3 muscarinic receptor on the acinar cells of the salivary glands, which links to elevation of intracellular calcium and subsequent secretion of salivary fluid [35, 36]. Together, measuring the salivary secretion, including the sAA contents and salivary flow, is able to reflect the sympathetic and parasympathetic nervous activities.

Considering that (1) UC patients present with altered ANS activities as well as psychological disorders, (2) the ANS dysfunction serves as one of the pathophysiological link between the psychological disorders and UC, and (3) salivary secretion is mainly regulated by ANS activity, we thus expected alterations of salivary secretion in UC patients, which might further support the altered ANS activities in the population.

## 2. Materials and Methods

### 2.1. Ethics Statement

The study was conducted adhering to the Declaration of Helsinki. Procedures involving human participants were reviewed and approved by the Academic Ethics Committee of Guangxi Traditional Chinese Medical University. A written informed consent was given by each participant.

### 2.2. Participants

The UC sample consisted 35 consecutively recruited patients with a confirmed diagnosis based on clinical, endoscopic, and histopathological evidence according to the standard diagnostic criteria [37]. These patients were receiving regular outpatient care at the Department of Gastroenterology, Ruikang Hospital of Guangxi Traditional Chinese Medical University. Thirty-two age- and sex-matched healthy controls were also enrolled from local advertisements. Psychological symptoms were evaluated by the Hospital Anxiety and Depression Scale (HADS) for each participant [38]. Individual who had HADS score more than 8 was recognized as suffering anxiety or depression. Structured clinical interviews were also performed. We excluded those who used psychotropic substances or painkillers within three months, because the drugs might significantly alter salivary secretion. Participants with alcohol abuse or suffered from recent oral or respiratory diseases were also excluded.

### 2.3. Collection of Saliva Samples

Saliva samples were collected in the morning. Participants were instructed not to eat or drink (except water) or do exercise before collection. For collection of basal saliva sample, we used the passive drooling method as described by Navazesh [39]. We then used citric acid to collect stimulated saliva as previously reported [40]. Sample volume (ml) as well as time used (min) were recorded for calculation of salivary flow rate (SFR; ml/min). Saliva samples were centrifuged at 12000*g* for 15 min, followed by collection of the supernatant and the remaining precipitate. The supernatant was subjected to determination of sAA amount (*μ*g/ml), while the precipitate (containing cheek cells) was used for *AMY1* gene copy number analysis.

### 2.4. sAA Amount Determined by Immunoblotting

sAA amount (*μ*g/ml) was determined by Western blotting. Briefly, saliva samples of equal quantity of total protein were prepared by solubilizing with the SDS-PAGE loading buffer and heating at 100°C for 10 min. For quantification purpose, a human sAA protein sample (Sigma-Aldrich) of known quantity was also run on each gel. Proteins were separated by SDS-PAGE and transferred onto a nitrocellulose membrane. The membrane was then blocked by 5% milk at room temperature, followed by incubation with rabbit anti-sAA antibody (Abcam) at 4°C overnight. After washing with PBST, the membrane was subjected to incubation with goat anti-rabbit IgG-horseradish peroxidase conjugate (R&D Systems) at room temperature for 1 hour. After washing, the membrane was then exposed to Pierce™ ECL Western Blotting Substrate (Thermo Scientific) for 1 min. A ChemiDoc XRS^+^ Chemiluminescence system (Bio-Rad) was used for detecting the sAA protein band. The test sAA amount (*μ*g/ml) was estimated by comparing with the respective standard sAA protein of known quantity.

### 2.5. DNA Extraction and Quantitative PCR for the *AMY1* Gene Copy Number

Genomic DNA was extracted from cheek cells by using DNAiso Reagent (Takara). Quantitative PCR (qPCR) was performed to determine the diploid *AMY1* gene copy number. Briefly, a fragment from tumor protein p53 (*TP53*) gene was amplified as an internal control. Primers for amplifications of *AMY1* and *TP53* were provided by Perry et al. [27]. Determination of DNA concentration, preparation of PCR mix, and thermal cycling were conducted according to a previous report [40]. *AMY1* diploid copy number was calculated by using a standard curve constructed from a reference DNA sample (NA18972; Coriell Cell Repositories) [27].

### 2.6. Statistical Analyses

Graph preparations as well as the statistical analyses were carried out by GraphPad Prism 5 (GraphPad Software). Data were expressed as mean ± standard deviation (SD), otherwise indicated. Unpaired Student's *t*-test was applied to compare means between groups for age, SFR, sAA amount, and *AMY1* gene copy number. A chi-square test was used to compare sex composition and the prevalence rate of anxiety and depression between the two study groups. A *p* value of less than 0.05 was considered as statistically significant.

## 3. Results

### 3.1. Characteristics of Participants

In the current study, we enrolled 35 UC patients as well as 32 healthy controls. Difference of age or sex (*χ*^2^) was not detected between the two study groups (Table 1). In the UC population, duration of the disease was 6.1 ± 2.3 years. Interestingly, we detected a significantly higher prevalence of psychological disturbances in the UC patients than controls (anxiety: *p* < 0.001; depression: *p* < 0.001), which was consistent with previous reports [4, 7].

### 3.2. Comparable Salivary Fluid Secretion between UC Patients and Controls

**Figure 1:**
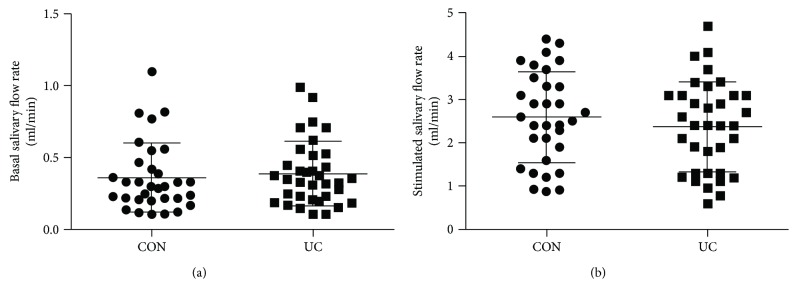
Comparable salivary fluid secretion between controls and UC patients. (a) Comparison of the basal SFR (ml/min) between controls (CON; *n* = 32) and UC patients (*n* = 35). (b) Comparison of the stimulated SFR. Values are means, with SD represented by vertical bars. No significant difference was obtained for basal or stimulated SFR between the study groups.

For basal SFR, we found extensive variations, ranging from 0.11 ml/min to 1.1 ml/min and 0.11 ml/min to 0.99 ml/min in the controls and UC patients, respectively (Figure 1(a)). After gustatory stimulation, salivary secretion robustly increased. We also detected extensive variations for the stimulated SFR, ranging from 0.88 ml/min to 4.4 ml/min and 0.59 ml/min to 4.7 ml/min in the controls and UC patients, respectively (Figure 1(b)). However, no difference was detected for basal or stimulated SFR between the two groups (basal: *t* = 0.466, *p* = 0.643; stimulated: *t* = 0.844, *p* = 0.402).

### 3.3. Elevated sAA Secretion in UC Patients

**Figure 2:**
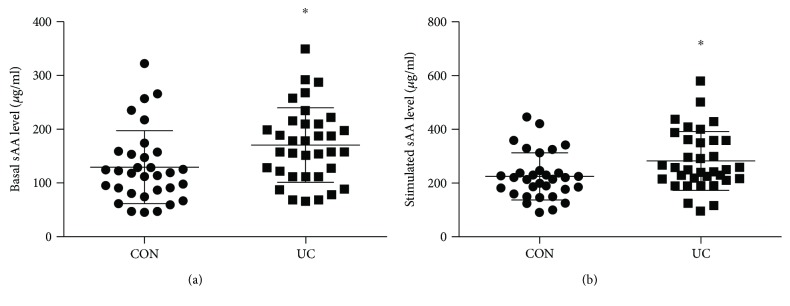
Elevated sAA secretion in UC patients relative to controls. (a) Comparison of the basal sAA level (*μ*g/ml) between controls (*n* = 32) and UC patients (*n* = 35). (b) Comparison of the stimulated sAA level. Values are means, with SD represented by vertical bars. ^∗^*p* < 0.05, UC patients versus controls.

We next determined the sAA level for each participant by immunoblotting. Standard sAA protein of known quantity enabled us to measure the sAA level (*μ*g/ml). Similar to salivary fluid secretion, we observed extensive variations for the basal sAA level, ranging from 46 *μ*g/ml to 322 *μ*g/ml and 66 *μ*g/ml to 350 *μ*g/ml in the controls and UC patients, respectively (Figure 2(a)). Similar variations were obtained for the stimulated sAA level, which varied from 89 *μ*g/ml to 445 *μ*g/ml and 96 *μ*g/ml to 580 *μ*g/ml in the controls and UC patients, respectively (Figure 2(b)). It is noteworthy that the sAA content robustly increased after gustatory stimulation within the group. Interestingly, we detected a higher level of basal sAA (*t* = 2.504, *p* = 0.015) as well as a higher level of stimulated sAA (*t* = 2.376, *p* = 0.021) in the UC population relative to controls (Figures 2(a) and 2(b)), which might implicate an elevated sympathetic nervous activity in the UC patients.

### 3.4. Similar *AMY1* Gene Number Copies between UC Patients and Controls

**Figure 3:**
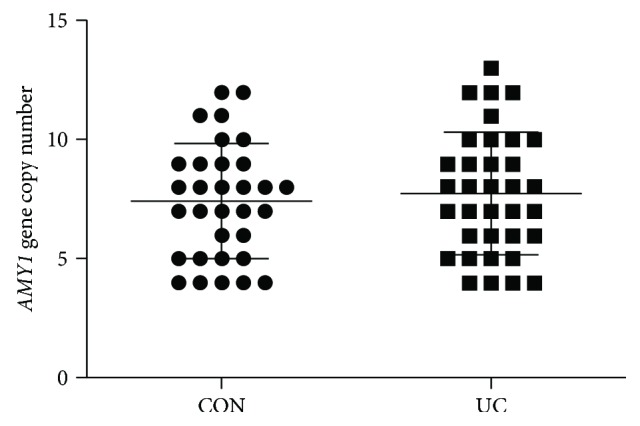
Similar *AMY1* gene copy number between controls and UC patients. Values are means, with SD represented by vertical bars. No significant difference was observed between the study groups.

The difference of the sAA level may be explained by *AMY1* gene copy number variations [27, 28]; we next determined the *AMY1* gene copy number for each participant by qPCR. As shown in Figure 3, we found that *AMY1* gene copy number for controls and UC patients varied from 4 to 12 and 4 to 13, respectively. No difference of the gene copy number was detected between the two study groups (*t* = 0.555, *p* = 0.581), which indicated that the *AMY1* gene copy number variations might not contribute to the altered sAA secretion in UC patients.

## 4. Discussion

In the current study, we collected 35 UC patients and 32 age- and sex-matched healthy controls. The psychological disorders (anxiety and depression), salivary fluid secretion, sAA level, and sAA gene (*AMY1*) copy number were evaluated/determined accordingly. In the UC population, we detected significantly higher prevalence of anxiety as well as depression compared to controls. Interestingly, we observed elevated basal and stimulated sAA levels in UC patients relative to controls. However, no difference was found for SFR before and after citric acid stimulation between the two groups. Besides, *AMY1* gene copy number was comparable between UC patients and controls. Our findings might implicate an altered ANS activity in UC patients, which was consistent with previous studies [15–17].

The ANS activity plays an important role in mediating the intestinal inflammation [11]. It is suggested that the sympathetic nervous activity has proinflammatory properties [12], while the parasympathetic division has anti-inflammatory properties [13]. Prospective studies linking the ANS abnormalities to severity of inflammation or other objective parameters reflecting UC disease activity would potentially have important implications for a better understanding of the pathogenesis of UC, which may in turn incubate potential development of new treatments for UC. In humans, the attempts to identify whether ANS alterations are present in UC has not come to a conclusion on the relationship between ANS function and UC disease activity. Nevertheless, a couple of previous studies had demonstrated altered ANS activities in UC patients [15–17], especially an elevated sympathetic nervous activity in this population [15, 16]. In the current study, we detected an elevated sAA secretion before and after gustatory stimulation, which also indicated an elevated sympathetic nervous activity in UC patients. Amazingly, there was a study demonstrating that the ANS abnormalities in UC were mainly attributed to dysfunction of the parasympathetic nervous activity [41]. However, our findings of comparable salivary fluid secretion between UC patients and controls did not support such result.

As aforementioned, the pathogenesis of UC is associated with psychiatric disturbances [5–7], while it has not come to a conclusion on the causal link between them [8–10]. We argued the possibility of a reciprocal causation between them. At the onset of the disease, the psychological stress is likely to emerge as a consequence of disease activity due to the recurrent and intractable characteristics of UC. After development of the disease, the psychological stress is able to, in turn, exacerbate the disease course. It brought to our attention that the psychological disturbances, anxiety and depression, are frequently associated with the altered ANS activity and that the ANS dysfunction serves as one pathophysiological link between the psychological disorders and a spectrum of diseases [18–23], including UC [24]. In the current study, the duration of UC disease was 6.1 years that might indicate a developed stage that the patients were at. And, we detected apparently higher prevalence of anxiety as well as depression in the UC population relative to control, which was consistent with previous reports [4, 7] and might partially explain the altered ANS dysfunction. Therefore, the chain of evidence/argument is likely to be “high prevalence of psychological disorders in patients with developed UC results in the ANS dysfunction (manifested by altered sAA secretion), which in turn promotes the UC pathogenesis”.

There is concern that salivary response, for example, the ratio of stimulated to the basal sAA level, may probably be better parameter reflecting ANS activity. In the present study, however, we did not detect any difference of sAA ratio or SFR ratio between the two study groups (data not shown). We argued that the basal sAA level is capable of reflecting the synthetic capacity of the salivary glands, while the stimulated sAA level is more likely to test the sympathetic nervous tension/sensitivity to stimulation. In the present study, UC patients presented higher basal and stimulated sAA levels relative to control, both pointing to an overactivity of the sympathetic nervous system due to the fact that the sAA synthesis as well as its secretion is tightly controlled by the sympathetic nervous system [31].

Because sAA production is mainly determined by the *AMY1* gene copy number [26], we thus detected whether difference of the gene copy number could be found between UC patients and healthy controls. However, no apparent difference was detected between them, which excluded the possibility of *AMY1* gene copy number variations in the pathogenesis of UC. Interestingly, we observed extensive variations of the *AMY1* gene copy number within each group, which was consistent with previous studies [28, 40]. Moreover, the relatively higher *AMY1* gene copies in the current population might suggest that a significant proportion of the participants' ancestors may undergo positive selection for increased *AMY1* gene copy when they traditionally fed on starch-based foods.

Salivary secretion can be affected by a myriad of factors, which has been largely elucidated [25]. Thus, the present study had attempted to exclude potential factors by giving instructions for each participant. Nevertheless, findings of our study should be taken as preliminary until replicated experiments with larger sample size are performed.

In conclusion, we detected an elevated sAA secretion in UC patients relative to controls, which might implicate sympathetic overactivity in the UC population. Together with previous studies, our findings corroborate the importance of ANS dysfunction in the pathogenesis of UC.

## Figures and Tables

**Table 1:** Characteristics of participants.

Characteristics	Control	UC	*p*
Number of participant	32	35	n.s.
Age (yr)	31 ± 8.8	29 ± 8.2	n.s.
Sex (female, male)	15, 17	18, 17	n.s.
Duration of UC (yr)	—	6.1 ± 2.3	—
Psychiatric disorder			
Anxiety (%)	6.3	54.3	^∗∗∗^
Depression (%)	0	25.7	^∗∗∗^

^∗∗∗^ *p* < 0.001 (chi-square test).

## Data Availability

Data are available upon reasonable request.
